# Cell-targeted PD-1 agonists are potent NK-cell inhibitors

**DOI:** 10.3389/fimmu.2025.1640509

**Published:** 2025-08-22

**Authors:** Harriet C. Pope, Ana L. Chiodetti, Alice Newey, Guillaume Rieunier, David X. Overton, Eduardo Mateos-Diaz, Tara M. Mahon, Giovanna Bossi, Hussein Al-Mossawi, Charlotte Viant

**Affiliations:** ^1^ Immunocore Ltd., Abingdon, United Kingdom; ^2^ Bath University, Bath, United Kingdom

**Keywords:** cell-targeted PD-1 agonists, NK cell, type 1 diabetes, autoimmune diseases, NK cell inhibition, PD-1 pathway

## Abstract

**Background:**

The programmed cell death protein 1 (PDCD1 or PD-1) is a key regulatory immune checkpoint and a major target for therapeutic intervention. In oncology, antibodies blocking the PD-1 pathway are used to activate immune cells to promote anti tumour immunity while in immune-mediated inflammatory diseases, PD-1 agonist molecules have the potential to achieve immune suppression. NK cells are a specialised population of innate lymphocytes able to recognize a large range of distressed cells including damaged tissues in autoimmune and inflammatory conditions. Of note, NK cells can upregulate PD-1 expression upon activation and their effector functions can be modulated by the PD-1 signalling pathway.

**Methods:**

We have generated a novel bispecific inhibitory molecule, comprised of a targeting domain highly specific for a pre-pro-insulin peptide presented by the HLA-A*02 molecules on the cell surface of pancreatic β-cells and a PD-1 agonist effector domain. Suppressive effects of the β-cell tethered bispecific PD-1 agonist molecule on NK cells and NK92-PD-1 cell line activation were assessed through gene expression, cell surface expression of the CD107a degranulation marker, intracellular IFNγ production and Granzyme B secretion. EndoC-b cells proliferation and insulin production were also measured.

**Results:**

We observed that the bispecific PD-1 agonist molecules tethered to pancreatic b-cells accumulate at the immunological synapse, modify NK cell gene expression and decrease their inflammatory and cytotoxic functions.

**Conclusions:**

Targeted PD-1 agonist molecules, inhibiting T cells and NK cells in a tissue-specific manner offer a new promising treatment for autoimmune and inflammatory diseases.

## Introduction

Natural killer (NK) cells are cytotoxic innate lymphocytes (1), responding to virally infected and transformed cells (2–5). NK cells provide a protective role in tumour surveillance and control of infections, but they are also involved in the onset, maintenance, or progression of several immune-mediated inflammatory diseases (6–17).

NK cells express a range of inhibitory receptors either constitutively or upon activation, including the immune checkpoint molecule PD-1 (18, 19). PD-1^+^ NK cells can be detected in healthy donors’ peripheral blood (20–23), and PD-1^−^ NK cells contain a pool of cytoplasmic PD-1 protein compatible with rapid surface expression upon stimulation (21, 24). Upregulation of NK-cell PD-1 expression has been described in several cancer settings (20, 25–31), in addition to viral and parasite infections such as HIV (32), hepatitis C (33), or malaria (34). In autoimmune diseases, NK PD-1 expression has been observed in systemic lupus erythematosus (SLE) (35), autoimmune thyroid disease (36), and models of type I diabetes (T1D) (37). PD-1^+^ NK-cell activation is downregulated upon PD-1/programmed death-ligand 1 (PD-L1) interaction, and PD-L1-mediated NK-cell inhibition can be reversed by PD-1/PD-L1 blockade in human *in vitro* studies and mouse models (18, 28, 29, 31, 38–41). NK-cell effector functions are potentiated in the context of increased PD-1 blockade; however, the effect of a PD-1 agonist therapy on NK-cell activation is not well understood.

We generated a novel bispecific, targeted molecule named immune modulating monoclonal T-cell receptor (TCR) against autoimmunity (ImmTAAI). The targeting domain is highly specific for a peptide presented by HLA-A*02 molecules on the targeted tissues, and the effector portion comprises a PD-1 agonist molecule (**Supplementary Figure S1A**). These molecules are potent inhibitors of T-cell function (42); in addition, they have the potential to inhibit other immune cells expressing PD-1.

T1D is a multifactorial autoimmune disease in which pancreatic β islets are destroyed by the immune system. To protect these insulin-producing cells, our novel ImmTAAI contains a TCR targeted to pre-pro-insulin peptide (PPI_15–24_, PPI-ImmTAAI) specifically presented by pancreatic β cells (43). PPI-ImmTAAI inhibits T-cell TCR signalling and suppresses T-cell cytokine production and cytotoxicity *in vitro* (42). Whilst T1D is considered an autoimmune disease driven primarily by autoreactive T cells, there is increasing evidence that innate immune cells contribute to the pathogenesis (44). NK cells have been detected in the pancreas of T1D patients (45) and in T1D mouse models (37, 46, 47). Human pancreatic β cells activate NK-cell cytotoxic and inflammatory function *in vitro* (37, 44, 48–51), and in mouse models, NK-cell depletion protects against T1D development (37, 46, 47).

Here, we studied the effect of a targeted PD-1 agonist on NK-cell function using *in vitro* activation of an NK-cell line and primary NK cells with PPI-ImmTAAI tethered to target cells, and demonstrate a functional role of NK PD-1 modulation in the inflammatory context.

## Methods

### Cell lines and lentiviral transduction

The NK92 cell line was obtained from the Basel Institute for Immunology and maintained in GMP SCGM medium (20802-0500, Sartorius Stedim, Gottingen, Germany) supplemented with 20% heat-inactivated fetal bovine serum (FBS), 50 U/mL penicillin and 50 µg/mL streptomycin (pen/strep, 15070-063, Gibco, Thermo Fisher Scientific Waltham, Massachusetts, US), and IL-2 (500 U/mL, 200-02, Peprotech, Cranbury, New Jersey, US).

The K562 cell line was maintained in R10 growth media: Roswell Park Memorial Institute 1640 medium (RPMI-1640, 42401-018, Gibco) supplemented with 10% heat-inactivated FBS, l-glutamine (2 mM, 25030-024, Gibco), and pen/strep (50 U/mL penicillin and 50 µg/mL strep, 15070-063, Gibco).

The EndoC-β cell line BH2 was in-licensed from Human Cell Design, Toulouse, France and maintained in Optiβ1 media (OB1-100, Human Cell Design) in tissue culture vessels precoated with β coat (BC-120, Human Cell Design).

The NHDF cell line was purchased from Lonza (CC-2511) and maintained in FBM-2 medium (CC-3132, Lonza, Basel, Switzerland).

NK92 and primary NK cells were transduced with PDCD1 (PD-1), K562 were transduced with HLA-A2 β2-microglobulin and CD274 (PD-L1), and EndoC-β cells were transduced with HLA-A2 β2-microglobulin and IncuCyte NucLight Red Lentivirus Reagent (NucLight red).

Lentiviral vectors containing PDCD1 (PD-1) and CD274 (PD-L1) were obtained from OriGene (Rockville, Maryland, US, RC210364L1 and RC213071L1, respectively). NucLight red was obtained from Sartorius, Göttingen, Germany. Lentiviral vectors for HLA-A2/β2-microglobulin were produced in-house.

### Human NK cells

NK cells were isolated from healthy volunteer PBMCs by negative isolation (NK Cell Isolation Kit, 130-092-657, Miltenyi, Bergisch Gladbach, Germany) according to the manufacturer’s protocol. Isolated NK cells were preactivated in R10 growth media supplemented with IL-2 (1,000 U/mL, 200-02, Peprotech) for 5–7 days prior to activation with EndoC-β cells or cultivated in NK MACS medium (130-112-968, Miltenyi) with IL-15 (140 U/mL, 200-15, Peprotech) and IL-2 (500 U/mL, 200-02, Peprotech) prior to lentiviral transduction with PD-1.

### NK92/K562-HLA-A*02 *in vitro* assays

K562-HLA-A*02 target cells were labelled with Cell Tracker Orange (CTO; C34551, Invitrogen, Thermo Fisher Scientific, Carlsbad, California, US) or Violet (CTV; C10094, Invitrogen) according to the manufacturer’s protocol. K562-HLA-A*02 were then loaded with PPI peptide (final concentration: 20 µM, ALWGPDPAAA) for 1 h at 37°C. Target cells were then preincubated with PPI-ImmTAAI or control-ImmTAAI at the indicated final concentration for 1 h at 37°C before adding the effector cells.

#### Short activation

NK92-PD-1 cells were incubated with K562-HLA-A*02-PPI/ImmTAAI molecules target cells for 4 h at a 1:4 effector-to-target (E:T) ratio (except if indicated otherwise). NK92-PD-1 activation was assessed by measuring the cytokine concentration in the culture supernatant or by the expression of CD107a and intracellular IFN-γ when the cells were activated in the presence of anti-CD107a-APC (328620, 1/100, BioLegend, San Diego, CA, US) and monensin and brefeldin A (GolgiPlug 1/500, 555029 and GolgiStop, 1/750, 554724, BD Bioscience, Franklin Lakes, New Jersey, US).

#### Long activation

NK92-PD-1 cells were incubated with K562-HLA-A*02-PPI/ImmTAAI molecules target cells for 20 h at a 1:4 or 1:2 E:T ratio. NK92-PD-1 activation was assessed by measuring cytokines and granzyme B concentration in the culture supernatant, by analysing the killing of the target cells stained with propidium iodide, and or by sequencing sorted NK92-PD-1 mRNA.

### NK92/NKp30 plate-bound activation

NK92-PD-1 were activated in a 96-well plate coated with 30 µg/mL anti-NKp30 (AF1849, Bio-Techne Ltd., Minneapolis, Minnesota, US) for 4 h in R10 medium in the presence of anti-CD107a-APC (328620, 1/100, BioLegend) and monensin and brefeldin A (GolgiPlug 1/500, 555029 and GolgiStop, 1/750, 554724, BD Bioscience) along with the indicated concentration of PPI-ImmTAAI, ctr-ImmTAAI, or anti-PD-1 agonist (rosnilimab, pM-range affinity, AnaptysBio).

### NK92 or primary NK cell/EndoC-β-cell *in vitro* assays

EndoC-β-cell (BH2-HLA-A*02, NucLight red) were plated overnight in precoated plates, then preincubated with PPI-ImmTAAI or ctr-ImmTAAI at the indicated concentration for 1 h at 37°C before adding the effector cells.

#### Short activation

NK or NK92-PD-1 cells were incubated with EndoC-β-cell/ImmTAAI molecule target cells for 4 h at a 1:2 E:T ratio. NK92-PD-1 or NK cells activation was assessed by measuring cytokine concentrations in the culture supernatant or by the expression of CD107a and intracellular IFN-γ when the cells were activated in the presence of anti-CD107a-APC (328620, 1/100, BioLegend), monensin and brefeldin A (GolgiPlug 1/500, 555029 and GolgiStop, 1/750 554724, BD Bioscience).

#### Long activation

NK92-PD-1 cells were incubated with EndoC-β-cell/ImmTAAI molecules target cells for 70 h at a 1:1 or 1:2 E:T ratio. Cell killing was determined by quantification of EndoC-β-cell number over time using the IncuCyte S3 imaging system (Sartorius Stedim). The number of red nucleus-labelled cells at each time point was normalised to the initial number of objects to account for variation in cell density within the area visualised. The number of events was acquired from five images and averaged. NK92-PD-1 activation was assessed by measuring cytokine concentrations in the culture supernatant after 70 h.

### NHDF fibroblast activation

NHDF were cultured in FBM-2 medium (CC-3132, Lonza). Supernatant from activated NK92-PD-1 was added at a 1/20 dilution. Supernatants from activated fibroblasts or control conditions were collected after 20 h. CXCL1 production was analysed by enzyme-linked immunosorbent assay (ELISA; DY275-05, Bio-Techne Ltd.).

### Glucose-stimulated insulin production by EndoC-β cells

After performing an EndoC-β cell killing assay with NK92-PD-1 for 70 h in the presence of different ImmTAAI molecule concentrations, the culture medium was carefully removed and replaced with Ulti-ST starvation medium (UST-50-BSA, Human Cell Design) for 24 h. Cells were washed and incubated with βKrebs medium (BK-250, Human Cell Design) for 1 h, and then with 20 mM glucose (G7021, Merck, Darmstadt, Germany) βKrebs medium for 1 h. The supernatant was collected, and insulin was measured by ELISA (R&D Systems, DY8056-05).

### Cytokine and granzyme B secretion

Concentrations of IFN-γ, TNF-α, and GM-CSF from the supernatants of activated NK92-PD-1 and NK-cell cultures, collected at the indicated time point, were measured by flow cytometry using LEGENDplex Human Tuberculosis Panel 1 kit (741397, BioLegend), according to the manufacturer’s protocol. The concentrations of granzyme B and CXCL1 from the supernatant of NK92-PD-1 and NHDF cultures were collected at the indicated time point and measured by ELISA (DGZB00 and DY275-05, Bio-Techne Ltd.), according to the manufacturer’s protocol.

### Flow cytometry

Samples were collected in fluorescence-activated cell sorting (FACS) buffer (phosphate-buffered saline [PBS] 1 ×, 10% fetal calf serum [FCS], 2 mM ethylenediaminetetraacetic acid [EDTA]). Cells expressing Fc-receptors were incubated with 5 mg/mL of Fc block (422302, BioLegend) for 15 min at 4°C in FACS buffer. Cell surface antigens were stained for 30 min at 4°C in FACS buffer. Foxp3 intracellular staining kit was used to perform intracellular staining according to the manufacturer’s protocol (00-5523-00, eBioscience, San Diego, California, US). Dead cells were stained with propidium iodide for 15 min at 4°C in FACS buffer (5 µg/mL, P4170, Sigma).

Flow cytometric analysis was performed on a BD Fortessa-X20 using the following antibodies: from BD Bioscience—anti-CD56-BV711 (563169), anti-IFN-γ-BV650 (563416), anti-PD-1-PE-Cy7 (561271), anti-NKp30-PE (558407), anti-CD56-BB700 (566400), anti-CD3-A647 (560626), anti-PD-1-BV421 (564323), and anti-CD56-AF488 (557699); from BioLegend—anti-NKp46-BV711 (331936), anti-CD107a-APC (328620), anti-PDL1-PE (329706), anti-CD33-BV421 (303416), and anti-human-Fc-PE (410708); and from Invitrogen—Aqua fluorescent reactive dye (L34957A).

### Number of molecules or ImmTAAI molecules per cell studies

K562-HLA-A*02-PPI and EndoC-β cells were incubated for 4 h with different concentrations of ctr- or PPI-ImmTAAI. ImmTAAI molecules binding to target cells were stained with a monoclonal anti-human-Fc-PE antibody (410708, BioLegend) for 30 min in FACS buffer (PBS 1 ×, 10% FCS, 2 mM EDTA). K562-HLA-A*02-PD-L1 and NK92-PD-1 cells were stained with anti-PD-L1-PE and anti-PD-1-PE for 30 min in FACS buffer. Numbers of PD-1, PD-L1, and ImmTAAI molecules were calculated with BD Quantibrite PE beads (340495, BD Bioscience) according to the manufacturer’s protocol.

### NK92-PD-1 RNA sequencing

NK92-PD-1 cells, activated or not with K562-HLA-A*02-PPI/ImmTAAI molecules for 20 h, were sorted with a Sony sorter SH800. NK92-PD-1 isolation was analysed by flow cytometry, and cell pellets containing one to two million cells were lysed with Buffer RLT Lysis buffer (350 µL, 1015762, QIAGEN, Hilden, Germany) and stored at – 80°C.

The RNA samples were submitted for paired-end RNA sequencing by GENEWIZ and sequenced on the Illumina Nextseq to a depth of ~ 20 million reads per sample. Following RNA sequencing, raw fastq files were trimmed with Trim Galore (v0.6.7) with the –nextseq-trim option enabled. Quality was then assessed using FastQC and MultiQC (v1.12) (90). Transcript mapping and quantification were performed using the QIAGEN OmicSoft Suite software, version 10.0.1.81, including the use of the QIAGEN Omicsoft Aligner (91). Downstream analysis of the resulting data was performed in R v4.4.1. BiomaRt v2.60.1 was used for gene name ID conversion. Differential expression was conducted using DESeq2 v1.44.0, controlling for experiment date as a batch effect. For differential expression, only genes with > 10 counts in at least three samples were considered. Genes were defined as significantly differentially expressed if they had a log2 fold change > 0.25 or ≤ 0.25, with an adjusted *p*-value < 0.05. fGSEA v1.30.0 was used for GSEA, with a subset of genes having a *p*-value < 0.1, then ranked by log2 fold change for the statistical test.

### Confocal microscopy of NK92/K562 immune synapse

K562-HLA-A*02 cells were stained with 1 µM Cell Tracker Deep Red Dye (C3455, eBioscience) for 20 min at 37°C according to manufacturer’s instructions. K562-HLA-A*02 cells were incubated for 1 h at 37°C with or without pre-pro-insulin (PPI_15–24_) peptide in R10 media. Both PPI-pulsed and unpulsed K562-HLA-A*02 cells were then incubated with PPI-ImmTAAI directly conjugated with AF488 for 1 h at 37°C in R10. Excess PPI-ImmTAAI was washed away, and K562-HLA-A*02 cells were resuspended in serum-free RPMI-1640. PPI-pulsed and unpulsed K562-HLA-A*02 cells were then seeded on an eight-well-high glass-bottom chamber (80807, Ibidi) at a concentration of 10^5^ cells/well. Cells were incubated for 20 min at 37°C to allow the target K562 cells to adhere to the bottom of the chamber. NK92 and NK92-PD-1 cell lines were maintained in CellGro SCGM (Cellgenix, Freiburg, Germany) media supplemented with 500 U/mL of IL-2 (PROELUKIN; Aldesleukin, Iovance Biotherapeutics, San Carlos, CA, US) and 20% FBS until use. On the day of the assay, NK92 cell lines were resuspended in CellGrow media without supplements and added separately to chambers containing either K562-HLA-A*02-PPI or K562-HLA-A*02 cells at 10^5^ cells/well to reach a 1:1 ratio with the target cells. Co-cultures were kept in an incubator at 37°C 5% CO_2_ for 15 min and then fixed with paraformaldehyde fixation solution (554655, BD) for 20 min at 4°C. The fixation solution was carefully removed and replaced with PBS buffer. DAPI was added to the well for counterstaining and washed off after 5 min. Images were acquired using a Nikon Eclipse Ti2 confocal microscope with a 100× oil immersion objective.

### Statistical analysis of confocal microscopy images

Images of PPI-loaded K562-HLA-A*02 with bound PPI-ImmTAAI AF488, co-cultured with NK92 on NK92-PD-1 cells, were analyse with open-source ImageJ software. Intensity plot profiles were calculated by tracing a line across the K562 cells in contact with NK92 cells and represent the mean fluorescence intensity (MFI) of the green channel corresponding to the PPI-ImmTAAI AF488 molecules. The ratio between the maximum MFI at the K562-NK92 contact point and the opposite side of each cell synapse was calculated and plotted as a scatter plot using GraphPad Prism version 10.

### PPI-ImmTAAI binding to human pancreatic β cells

Frozen HLA-A*02-positive and HLA-A*02-negative pancreatic and CRC tissues (embedded in optimal cutting temperature [OCT] blocks) were cryo-sectioned at − 20°C using a cryostat (Cryostar NX70, Thermo Scientific, Waltham, Massachusetts, US). OCT sections were cut at 10 µm and transferred to SuperFrost Plus™ microscope slides. Slides were briefly fixed in ice-cold acetone and used immediately. Each slide was appropriately barcoded with a hydrophobic slide label (Ventana Ebar) before being loaded into the Ventana auto-stainer (BenchMark Ultra, Roche). Sections were stained with 50 nM rabbit-Fc-conjugated PPI-ImmTAAI for 32 min at 37°C. The signal was developed using a secondary antibody, anti-rabbit coupled with HQ (760-4815, Roche, Basel, Switzerland), and amplified with a tertiary antibody anti-HQ coupled with a horseradish peroxidase (HRP) chromogen (760-4602, ChromoMap DAB 05266645001, Roche).

Dual staining was performed on tissue sections using rabbit-Fc-conjugated PPI-ImmTAAI, which was developed with a secondary antibody anti-rabbit coupled with NP (07425317001, Roche) and amplified with a tertiary antibody anti-NP coupled with an AP chromogen (07425325001, Roche). Slides were denatured for 24 min at 100°C and additionally stained with 10 ng/mL insulin primary antibody for 32 min at 37°C (HPA004932, Sigma Aldrich). The signal was developed using a secondary antibody, anti-rabbit coupled with HQ (760-4815, Roche), and amplified with a tertiary antibody anti-HQ coupled with an HRP chromogen (760-4602, Discovery Purple 07053983001, Roche).

The sections were counterstained with haematoxylin II (790-2208, Roche). Upon completion of the staining run, slides were loaded into racks for the cover-slipper. Slides were briefly washed in tepid soapy water and rinsed with distilled water to remove residual oil. They were then dehydrated by dipping through 70% ethanol, followed by three changes of 100% ethanol, cleared through two changes of xylene, and mounted and coverslipped with DPX on the CTM6 (Leica, Wetzlar, Germany) auto-coverslipper. Slides were air-dried in a fume hood overnight. To prepare the slides for scanning, excess DPX was removed by razor-scraping, and the slides were wiped clean with 100% ethanol, ready for visualisation using the Pannoramic slide scanner (3DHISTECH, Budapest, Hungary).

### Statistics

Statistical information, including the number of samples (*n*), number of experiments, mean (centre bars), and statistical significance values, is indicated in the figure legends. Statistical significance was determined with GraphPad Prism with the tests indicated in each figure. Data were considered statistically significant at ^*^ *p* ≤ 0.05, ^**^ *p* ≤ 0.01, ^***^ *p* ≤ 0.001, and ^****^ *p* ≤ 0.0001.

## Results

### Targeted PD-1 agonist molecules inhibit NK92-PD-1 cell line activation

**Figure 1:**
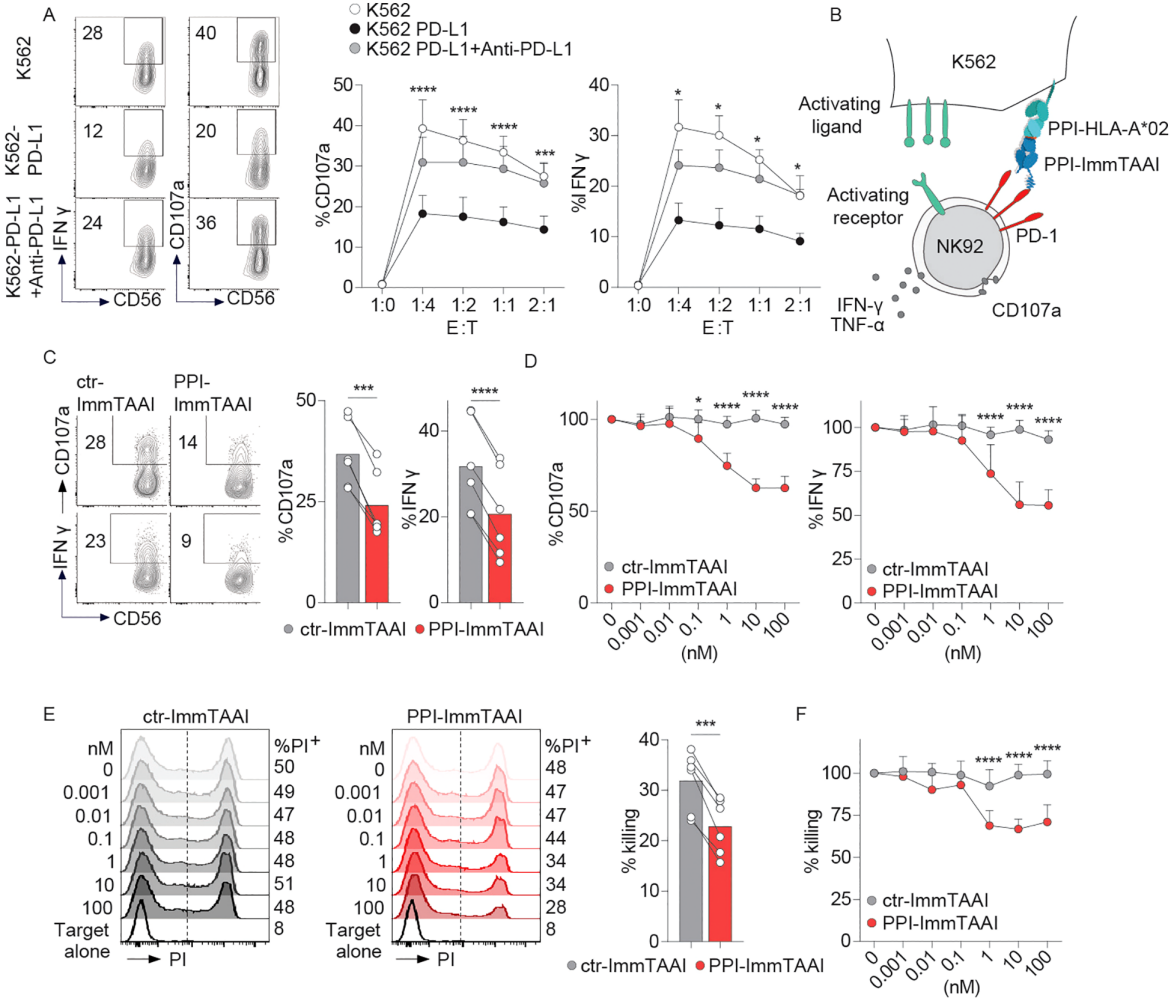
PPI-ImmTAAI inhibits NK92-PD-1 cells activated with K562-HLA-A*02-PPI. **(A)** Representative flow cytometry profile and graphs summarising CD107a expression and IFN-γ intracellular production by NK92-PD-1 cells without (left, white circles) or with anti-PD-L1 (right, black circles), activated using different ratios of K562 or K562-PD-L1 target cells. Each circle represents the mean (*n* = 6; three independent experiments; two-way ANOVA; SD; ^*^ *p* ≤ 0.05; ^***^ *p* ≤ 0.001; ^****^ *p* ≤ 0.0001). **(B)** Graphical representation of NK92-PD-1 activation with K562-HLA-A*02 cells loaded with the PPI_15–24_ peptide in the presence of PPI-ImmTAAI. **(C, D)** NK92-PD-1 cells were stimulated for 4 h with K562-HLA-A*02-PPI in the presence of ctr-ImmTAAI (grey) or PPI-ImmTAAI (red). **(C)** Representative flow cytometry profiles and graphs summarising CD107a expression and IFN-γ intracellular production in the presence of 10 nM ImmTAAI molecules. Each dot represents one sample (mean, *n* = 6; three independent experiments; paired *t*-test; ^***^ *p* ≤ 0.001; ^****^ *p* ≤ 0.0001). **(D)** Graph summarising CD107a expression and IFN-γ intracellular production in the presence of different ImmTAAI molecules, normalised to 0 nM ImmTAAI values. Each dot represents the mean of three independent experiments (*n* = 6; two-way ANOVA; SD; ^*^ *p* ≤ 0.05; ^****^ *p* ≤ 0.0001). **(E, F)** NK92-PD-1 cells were stimulated for 20 h with K562-HLA-A*02-PPI in the presence of ctr-ImmTAAI (grey) or PPI-ImmTAAI (red). **(E)** Representative flow cytometry profiles and graph (10 nM) summarising the proportion of dead cells (PI^+^ cells) in the presence of ImmTAAI molecules. Each dot represents one sample (mean; *n* = 6; three independent experiments; paired *t*-test; ^***^ *p* ≤ 0.001). **(F)** Graph summarising the proportion of dead K562-HLA-A*02-PPI target cells in the presence of different concentrations of ImmTAAI, normalised to 0 nM ImmTAAI values. Each dot represents the mean of three independent experiments (*n* = 6; two-way ANOVA; SD; ^****^ *p* ≤ 0.0001).

To analyse the effect of the targeted PD-1 agonist, PPI-ImmTAAI molecule on NK-cell activation, we first used the NK92 cell line (52) transduced with PD-1 lentivirus (NK92-PD-1, **Supplementary Figure S2A**). NK92-PD-1 cells were co-cultured for 4 h with K562 target cells with or without PD-L1 expression (**Supplementary Figure S2A**; **Figure 1A**). NK92-PD-1 activation was assessed through surface expression of the CD107a degranulation marker and intracellular interferon gamma (IFN-γ) production (**Figure 1A**). NK92-PD-1 activation was decreased in co-culture with K562 cells expressing PD-L1, and increased in the presence of an anti-PD-L1 antibody, demonstrating a functional PD-1 inhibitory pathway on NK92-PD-1 cells (**Figure 1A**).

To examine the effect of the cell-bound PD-1 agonist on NK92-PD-1 activation, we pulsed K562-HLA-A*02 target cells with the PPI peptide, allowing a highly specific binding of the PPI-ImmTAAI (**Supplementary Figure S2B**). NK92-PD-1 were co-cultured for 4 h with K562-HLA-A*02-PPI/ImmTAAI molecule-bound target cells (**Figure 1B**), and NK92-PD-1 activation was assessed by CD107a and IFN-γ expression (**Figures 1C, D**). The survival of the effector and target cells was not affected by ImmTAAI molecules binding to PD-1 or HLA-A*02-PPI (**Supplementary Figures S2C–E**). PPI-ImmTAAI significantly decreased NK92-PD-1 activation compared to a nontargeted ImmTAAI (ctr-ImmTAAI) (**Supplementary Figures S2F, G**; **Figures 1C, D**). In the presence of 10 nM ImmTAAI molecules, mean CD107a expression decreased from 37% to 24%, and IFN-γ from 32% to 20% (**Figure 1C**). There was a dose-dependent inhibition of NK92-PD-1 activation, starting at 0.1 nM PPI-ImmTAAI and reaching a 37% decrease in CD107a expression and a 45% in IFN-γ production at 10 nM PPI-ImmTAAI (**Figure 1D**).

To evaluate the effect of the PPI-ImmTAAI on the NK92-PD-1 cytotoxic function, the killing of K562-HLA-A*02-PPI target cells was assessed after 20 h of co-culture with NK92-PD-1. The ability of NK92-PD-1 to kill the target cells was reduced in the presence of 1 nM PPI-ImmTAAI and reached a 35% decrease at 10 nM (**Figures 1E, F**). Thus, the targeted PD-1 agonist, PPI-ImmTAAI, significantly suppressed NK92-PD-1 activation and protected target cells from NK-cell-mediated killing.

### Targeted PD-1 agonist inhibition of NK92-PD-1 suppresses the inflammatory environment and tissue damage

NK-cell activation in autoimmune diseases contributes to the inflammatory environment (8). NK cells produce cytokines and extracellular granzyme B (GzB), which cause inflammation and tissue damage (53) in the context of human pancreatic β-cell destruction in T1D (54–56). We analysed the NK92-PD-1 extracellular secretion of GzB and proinflammatory cytokines after a prolonged 20-h activation with K562-HLA-A*02-PPI target cells and the impact of the PPI-ImmTAAI on these inflammatory effector functions.

**Figure 2:**
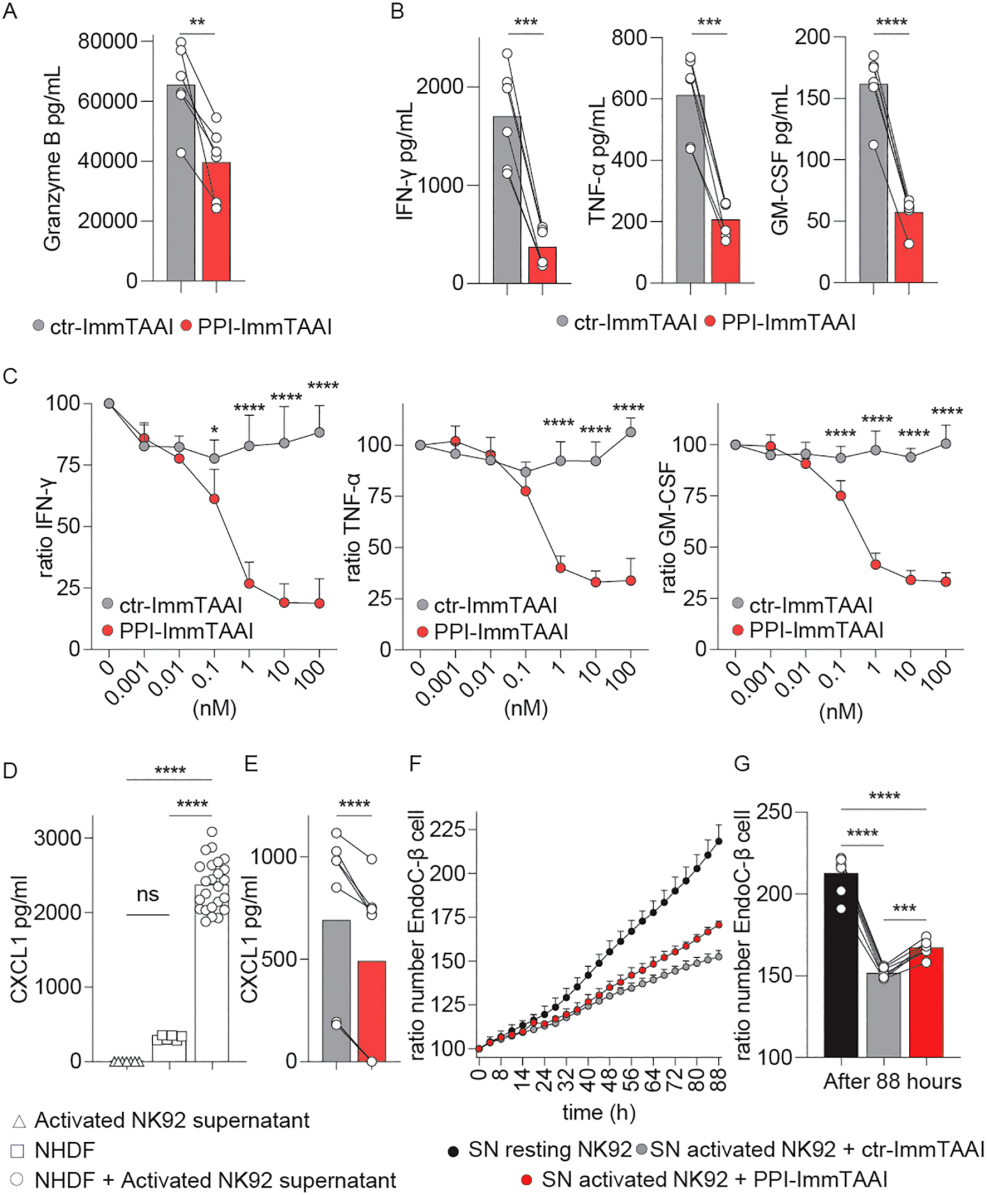
Effect of PPI-ImmTAAI on NK92-PD-1 inflammatory effector functions. **(A–G)** NK92-PD-1 cells were stimulated for 20 h with K562-HLA-A*02-PPI in the presence of ctr-ImmTAAI (grey) or PPI-ImmTAAI (red). **(A)** Graph summarising GrzB concentration in the culture supernatant in the presence of 10 nM ImmTAAI molecules. Each dot represents one sample (mean; *n* = 6; four independent experiments; paired *t*-test; **p ≤ 0.01). **(B)** Graphs summarising IFN-γ, TNF-α, and GM-CSF concentrations in the culture supernatant in the presence of 10 nM ImmTAAI molecules. Each dot represents one sample (mean; *n* = 6; three independent experiments; paired *t*-test; ***p ≤ 0.001; ****p ≤ 0.0001). **(C)** Graphs summarising IFN-γ, TNF-α, and GM-CSF concentrations in the culture supernatant in the presence of different ImmTAAI molecules, normalised to 0 nM ImmTAAI values. Each dot represents the mean of three independent experiments (*n* = 6; two-way ANOVA; SD; *p ≤ 0.05; ****p ≤ 0.0001). **(D)** Graph summarising CXCL1 concentration in the supernatants of activated NK92-PD-1 cells (triangles), NHDF cells (squares), or NHDF cells activated with NK92-PD-1 supernatant (circles). Each dot represents one sample (mean; *n* = 6–24; two independent experiments; one-way ANOVA; ****p ≤ 0.0001). **(E)** Graph summarising CXCL1 concentrations in the supernatant of NHDF cells stimulated with supernatants from activated NK92-PD-1/ctr-ImmTAAI cells (grey) or activated NK92-PD-1/PPI-ImmTAAI cells (red). Each dot represents one sample (mean; *n* = 6; three independent experiments; paired *t*-test; ****p ≤ 0.0001). **(F)** Graph representing the ratio of EndoC-β-cell number over time, normalised to 0 h and cultured in the presence of resting NK92-PD-1 supernatant (black), activated NK92-PD-1/ctr-ImmTAAI supernatant (grey), or activated NK92-PD-1/PPI-ImmTAAI supernatant (red) (representative of three independent experiments; SD). **(G)** Graph summarising the ratio of EndoC-β-cell number at 88 h, normalised to 0 h, and cultured in the presence of resting NK92-PD-1 supernatant (black), activated NK92-PD-1/ctr-ImmTAAI supernatant (grey), or activated NK92-PD-1/PPI-ImmTAAI supernatant (red). Each dot represents one sample (mean; *n* = 7; three independent experiments; one-way ANOVA; ***p ≤ 0.001; ****p ≤ 0.0001).

In the presence of PPI-ImmTAAI, GzB extracellular concentration significantly decreased from 65,442 to 39,537 pg/mL (average, **Figure 2A**). Moreover, PPI-ImmTAAI led to a broad decrease of inflammatory cytokine production by activated NK92-PD-1; 82% for IFN-γ (0.1 nM IC_50_), 67% for tumour necrosis factor alpha (TNF-α; 0.17 nM IC_50_), and 67% for granulocyte–macrophage colony-stimulating factor (GM-CSF; 0.16 nM IC_50_) (**Figures 2B, C**).

To further investigate the downstream effect of PPI-ImmTAAI on the inflammatory environment, we analysed the impact of the inflammatory milieu on a normal human dermal fibroblast (NHDF) cell line and a pancreatic-β (EndoC-β) cell line. NK92-PD-1 cells were activated for 20 h with K562-HLA-A*02-PPI target cells, and the supernatants were added to NHDF and EndoC-β cultures. We observed an increase in fibroblast CXCL1, a marker of activation (57), after co-culture with activated NK92-PD-1 supernatants (**Figure 2D**). A significant reduction of NHDF CXCL1 production was seen when the PPI-ImmTAAI was present during the NK92-PD-1 activation compared to a nontargeted ImmTAAI (**Figure 2E**, 490 pg/mL versus 690 pg/mL).

To understand the impact of inflammatory cytokines produced by the NK92-PD-1 cells on EndoC-β cells, we measured EndoC-β-cell growth for 88 h in the presence of supernatants from resting or activated NK92-PD-1 cells with control or PPI-ImmTAAI (**Figures 2F, G**). We observed decreased EndoC-β-cell growth with the activated NK92-PD-1 supernatant. This growth-inhibitory effect was reduced with supernatants from NK92-PD-1 cells activated in the presence of PPI-ImmTAAI (**Figures 2F, G**). Taken together, our data showed that PPI-ImmTAAI can decrease NK92-PD-1 cytokines and GzB production and support EndoC-β-cell proliferation whilst protecting against tissue damage.

### NK92-PD-1 inhibition is dependent on PPI-ImmTAAI localisation to the target: NK92-PD-1 cell interface

**Figure 3:**
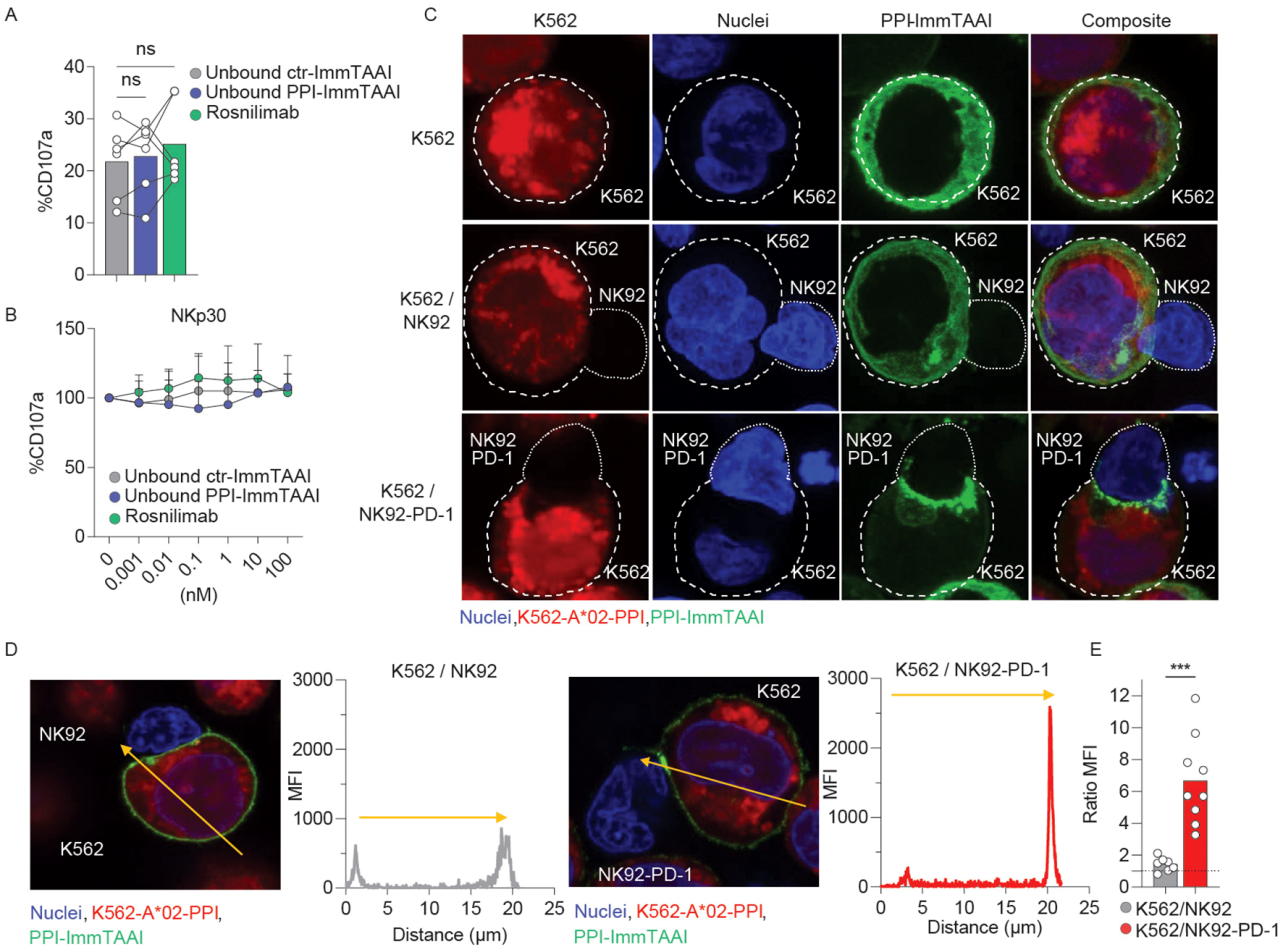
PPI-ImmTAAI crosslinked mode of action. **(A, B)** NK92-PD-1 cells were stimulated for 4 h with plate-bound NKp30 antibody in the presence of ctr-ImmTAAI (grey), unbound PPI-ImmTAAI (blue), or rosnilimab (anti-PD-1-agonist, green). **(A)** Graph summarising CD107a expression in the presence of 10 nM of ctr-ImmTAAI (grey), unbound PPI-ImmTAAI (blue), and rosnilimab. Each dot represents one sample (mean; *n* = 6; three independent experiments; one-way ANOVA). **(B)** Graph summarising CD107a expression in the presence of different molecule concentrations, normalised to 0 nM molecule values. Each dot represents the mean of three independent experiments (*n* = 6; two-way ANOVA; SD). **(C–E)** NK92 or NK92-PD-1 cells were co-cultured with K562-HLA-A*02-PPI/PPI-ImmTAAI AF488 for 15 min. **(C)** Representative 3D projections of PPI-ImmTAAI molecule localisation on confocal microscopy z-stack. Images show the localisation of PPI-ImmTAAI molecules (green) binding to the membrane of a K562-HLA-A*02-PPI cell (blue and red double positive, top), to a K562-HLA-A*02-PPI cell interacting with an NK92 cell (blue, middle), or to a K562-HLA-A*02-PPI cell interacting with an NK92-PD-1 cell (bottom) (two independent experiments). **(D)** Microscopy images and graphs summarising the intensity (MFI) of green fluorescence (PPI-ImmTAAI) across a K562-HLA-A*02-PPI (yellow arrow) interacting with an NK92 cell (left) or an NK92-PD-1 cell (right; two independent experiments, *n* = 7–9). **(E)** Graph summarising the ratio between the maximum MFI for the green channel (PPI-ImmTAAI) at the synapse site and the opposite site for each K562-HLA-A*02-PPI cell analysed, interacting with an NK92 cell (grey) or an NK92-PD-1 cell (red). Each dot represents one cell (mean; *n* = 7–9; two independent experiment; unpaired *t*-test; ^***^ *p* ≤ 0.001).

Contrary to systemic PD-1 agonist antibodies, the affinity of the effector arm of the PPI-ImmTAAI is in the nanomolar range. Thus, the ImmTAAI molecule exhibits preferential binding to the target cell because of its high-affinity TCR arm (picomolar range) (42). To understand if a high-affinity soluble untargeted PD-1 agonist can inhibit NK92-PD-1 activation in the absence of a crosslinking interaction with a target cell, we analysed the untargeted effect of the ImmTAAI molecules compared to a high-affinity anti-PD-1 agonist (rosnilimab, AnaptysBio, San Diego, CA, US, picomolar range affinity) on NK92-PD-1 activation (**Figures 3A, B**). In the absence of a crosslinking interaction, none of the molecules tested inhibited NK92-PD-1 activation with plate-bound NKp30 antibody (**Figures 3A, B**). Thus, systemic PD-1 agonist antibody molecules are unlikely to have an effect on NK-cell activation.

PD-1 molecules cluster at the synapse between NK92-PD-1 and target cells upon interaction with PD-L1, the natural ligand (28). To assess whether the PPI-ImmTAAI is able to localise to the NK92-PD-1/target cell interface, we conducted an immunofluorescence study with a fluorescently labelled PPI-ImmTAAI-AF488 (**Supplementary Figure S3**; **Figures 3C–E**). NK92-PD-1 were not labelled by PPI-ImmTAAI-AF488, and the molecule did not bind to K562-HLA-A*02 in the absence of PPI peptide pulsing (**Supplementary Figure S3**). We observed a homogeneous PPI-ImmTAAI-AF488 membrane staining on isolated K562-HLA-A*02-PPI (**Figure 3C**, top). This staining was not disrupted by K562-HLA-A*02-PPI interaction with PD-1-negative NK92 (**Figure 3C**, middle), as no difference was observed in AF488 fluorescence between the cell interaction site and the opposite side of the target cell membrane (**Figures 3D, E**). However, the PPI-ImmTAAI-AF488 accumulated at the NK92/K562 interface when NK92 expressed PD-1 (**Figure 3C**, bottom), as shown by a significant increase in AF488 fluorescence at the synapse formed by NK92-PD-1 and K562-HLA-A*02-PPI compared to the opposite side of the target cell membrane (**Figures 3D, E**). Thus, PD-1 inhibitory pathway activation required a targeted engagement and accumulation of the PD-1 agonist molecule at the target/NK92-PD-1 cell interface.

### PPI-ImmTAAI change activated NK92-PD-1 gene expression profile

To determine the broader effect of PPI-ImmTAAI on the transcriptional programme of activated NK92-PD-1 cells, we performed RNA sequencing (RNA-seq) on resting NK92-PD-1 cells and on purified NK92-PD-1 cells activated with K562-HLA-A*02-PPI in the presence of PPI-ImmTAAI or ctr-ImmTAAI (**Supplementary Figure S4**).

**Figure 4:**
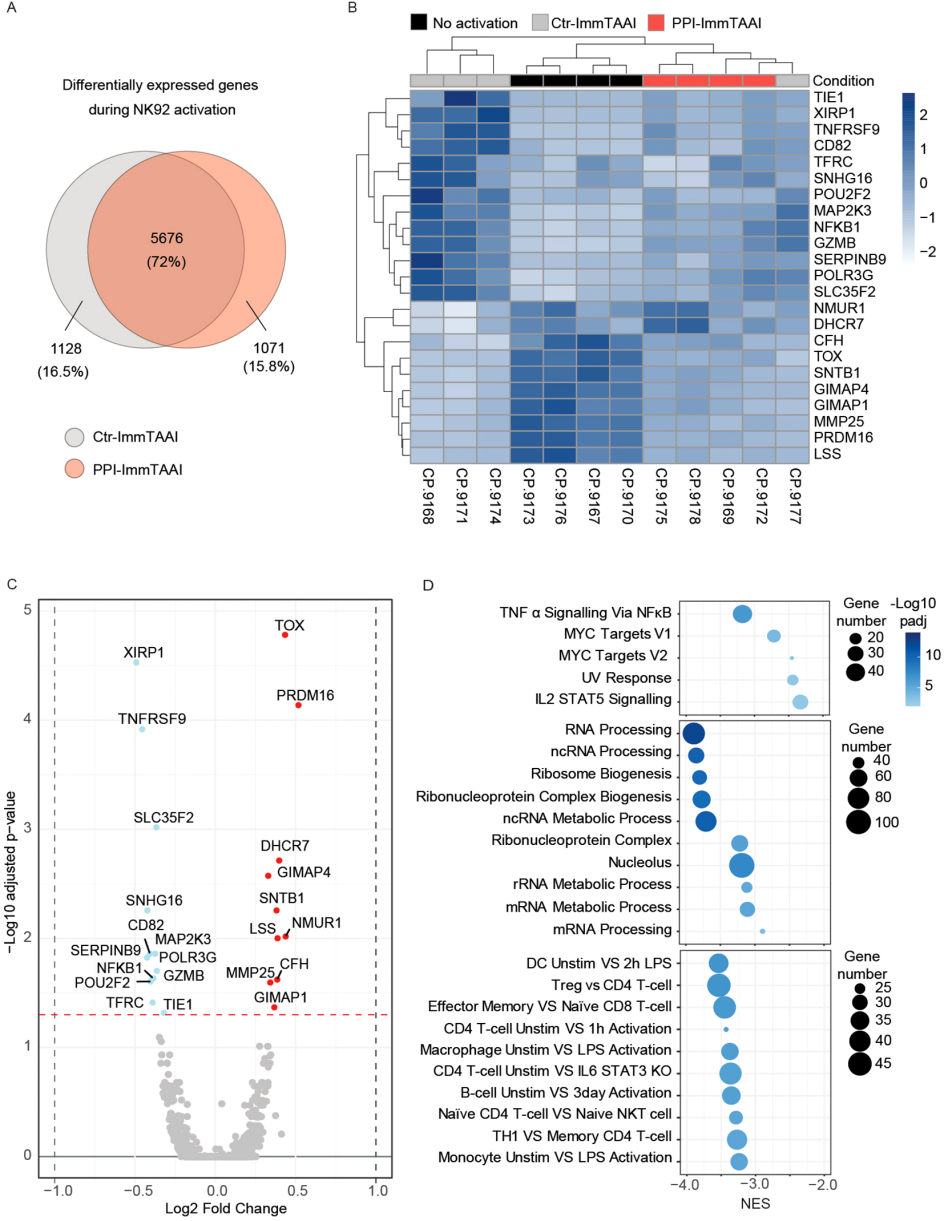
PPI-ImmTAAI effect on activated NK92-PD-1 gene expression. **(A–D)** Gene expression analyses of NK92-PD-1 cells that were activated (ctr-ImmTAAI: grey or PPI-ImmTAAI: red) or nonactivated (black) for 20 h with K562-HLA-A*02-PPI (four independent experiments). **(A)** Venn diagrams representing differentially expressed genes during NK92-PD-1 activation. Grey circles represent gene variations between resting NK92-PD-1 and NK92-PD-1 cells activated with ctr-ImmTAAI; red circles represent gene variations between resting NK92-PD-1 and NK92-PD-1 cells activated with PPI-ImmTAAI. **(B)** Heatmap of all significantly differentially expressed genes (adjusted *p*-value < 0.05) among nonactivated NK92-PD-1, activated NK92-PD-1 with ctr-ImmTAAI, or PPI-ImmTAAI. **(C)** Volcano plot showing the fold change in gene expression between activated NK92-PD-1 with ctr-ImmTAAI and activated NK92-PD-1 with PPI-ImmTAAI. Genes with an adjusted *p*-value < 0.05 were highlighted in colour; changes > log2 0.25-fold are highlighted in red, and changes < log2 − 0.25-fold are highlighted in blue. **(D)** Gene set enrichment analysis (GSEA) results showing the normalised enrichment scores (NES) for either all significantly enriched gene sets or the top 10 significant gene sets from the Hallmark, Gene Ontology, and Immunologic gene set collections, respectively. The number of genes in each gene set is represented by dot size, and −log10(*p*adj) is represented by the colour scale.

NK92-PD-1 activation led to a differential expression of 6,804 genes (grey circle, 3,164 upregulated and 3,640 downregulated genes) (**Figure 4A**). A similar number of genes were differentially expressed when NK92-PD-1 were activated in the presence of PPI-ImmTAAI (6,747genes; red circle, 3,166 upregulated and 3,581 downregulated genes). However, 15.8% of the differentially expressed genes were specific to NK92-PD-1 cells activated in the presence of the targeted PD-1 agonist PPI-ImmTAAI molecule (**Figure 4A**). We performed an unsupervised hierarchical clustering (DESeq) analysis based on differentially expressed genes between NK92-PD-1 activated cells in the presence of PPI-ImmTAAI or ctr-ImmTAAI. Samples activated in the presence of PPI-ImmTAAI clustered with the unactivated NK92-PD-1 cells and separately from three out of four NK92-PD-1 cell samples activated in the presence of ctr-ImmTAAI (**Figure 4B**). The gene expression differences between PPI-ImmTAAI- and ctr-ImmTAAI-treated cells were driven by the significant differential expression of 23 genes (**Figure 4C**). Specific key genes involved in NK-cell activation, such as TNFRSF9, CD82, MAP2K3, SERPINB9, GZMB, POU2F2, NFKB1, and TFRC (58–64), were downregulated in the presence of PPI-ImmTAAI compared to ctr-ImmTAAI (**Figure 4C**). Upregulated genes in the presence of PPI-ImmTAAI compared to ctr-ImmTAAI included TOX, a transcription factor highly expressed in immature NK cells (65), and MMP25, a matrix metalloproteinase which decreases antibody-dependent cellular cytotoxicity (ADCC) by downregulating CD16 expression (66). Gene set enrichment analysis (GSEA) revealed 93 pathways significantly downregulated in PPI-ImmTAAI-treated cells compared to NK92-PD-1 cells activated in the presence of ctr-ImmTAAI, including pathways involved in metabolism, transcription, proliferation, inflammation, and immune cell activation (67) (**Figure 4D**; **Supplementary Figure S4F**). Thus, PPI-ImmTAAI modulated a broad range of NK92-PD-1 genes and pathways involved in NK-cell activation and effector function.

### PPI-ImmTAAI inhibits NK92-PD-1 activation against EndoC-β target cells

**Figure 5:**
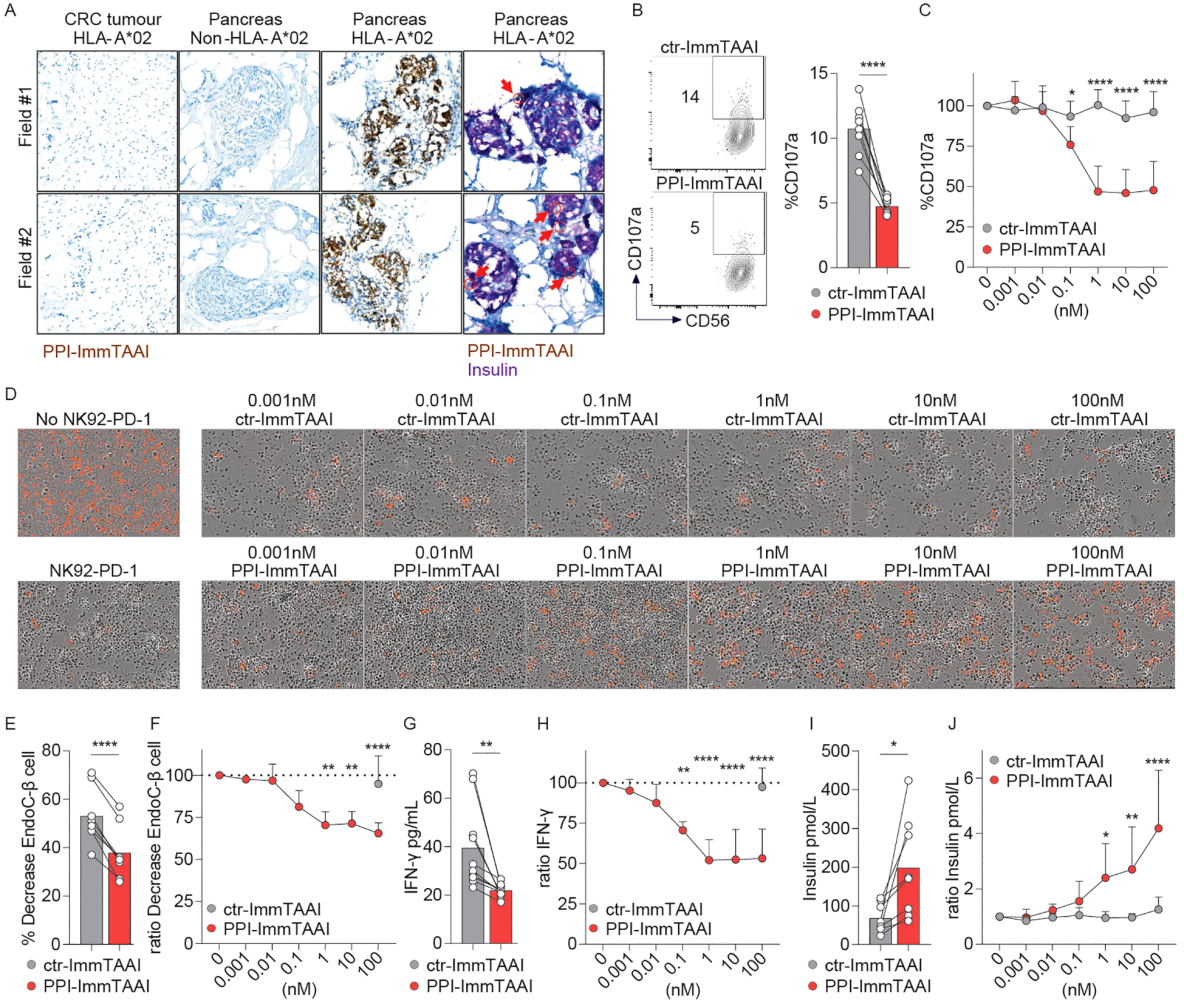
PPI-ImmTAAI inhibits NK92-PD-1 cells stimulated *in vitro* with EndoC-β cells. **(A)** Representative immunohistochemistry images of HLA-A*02 pancreatic tissues, where β cells are stained with PPI-ImmTAAI (brown and yellow, red arrows) and with insulin (purple), and of the relevant control tissues (β cells from non-HLA-A*02 pancreatic tissues and PPI-negative cells from HLA-A*02 CRC tissues). NK92-PD-1 cells were stimulated for 4 h **(B, C)** or 70 h **(D–G)** with EndoC-β cells in the presence of ctr-ImmTAAI (grey) or PPI-ImmTAAI (red). **(B)** Representative flow cytometry profiles and a graph summarising CD107a expression in the presence of 10 nM ImmTAAI molecules. Each dot represents one sample (mean; *n* = 8; three independent experiments; paired *t*-test; ^****^ *p* ≤ 0.0001). **(C)** Graph summarising CD107a expression in the presence of different concentrations of ImmTAAI molecules, normalised to 0 nM ImmTAAI values. Each dot represents the mean of three independent experiments (*n* = 6; two-way ANOVA; SD; ^*^ *p* ≤ 0.05; ^****^ *p* ≤ 0.0001). **(D)** Representative microscopy images of EndoC-β cell (red) cultured for 70 h alone (left top) or with NK92-PD-1 in the presence of different concentrations of ctr-ImmTAAI (top) or PPI-ImmTAAI (bottom; representative of three independent experiments). **(E, G)** Graphs summarising the decrease in the proportion of live target cells and the IFN-γ concentration in the culture supernatant, in the presence of 100 nM ImmTAAI molecules. Each dot represents one sample (mean; *n* = 8–10; four independent experiments; paired *t*-test; ^**^ *p* ≤ 0.01; ^***^ *p* ≤ 0.001). **(F, H)** Graphs summarising the decrease in the proportion of live target cells and the IFN-γ concentration in the culture supernatant in the presence of different concentrations of ImmTAAI molecules, normalised to 0 nM ImmTAAI values. Each dot represents the mean of four independent experiments (*n* = 8–10; mean; two-way ANOVA; SD; ^**^ *p* ≤ 0.01; ^****^ *p* ≤ 0.0001). **(I, J)** After 70 h of co-culture of NK92-PD-1 and EndoC-β cells, as described in **(D**, **E)**, the concentration of insulin secreted by the remaining EndoC-β cells following a glucose challenge was measured. **(I)** Graph summarising the insulin concentration in the culture supernatant when NK92-PD-1 cells were co-cultured with EndoC-β cells in the presence of 10 nM ImmTAAI molecules. Each dot represents one sample (*n* = 8; mean; five independent experiments; paired *t*-test; ^*^ *p* ≤ 0.05). **(J)** Graph summarising the insulin concentration in the culture supernatant when NK92-PD-1 cells were co-cultured with EndoC-β cells in the presence of different concentrations of ImmTAAI molecules and normalised to 0 nM ImmTAAI values. Each dot represents the mean of five independent experiments (*n* = 6; two-way ANOVA; SD; ^*^ *p* ≤ 0.05; ^**^ *p* ≤ 0.01; ^****^ *p* ≤ 0.0001).

The PPI-ImmTAAI molecule has been engineered to specifically bind human pancreatic β cells, as they are the only cell type to present the PPI_15–24_ peptide, allowing organ-specific inhibition of the immune system in the context of T1D treatment (**Figure 5A**). Histochemistry labelling of different human tissues showed that PPI-ImmTAAI specifically bound to pancreatic-β islets of HLA-A*02 donors and colocalised with insulin staining. No PPI-ImmTAAI staining was detected in pancreatic tissues from a non-HLA-A*02 donor or in an irrelevant control colorectal cancer sample from an HLA-A*02 donor (**Figure 5A**).

To functionally evaluate the PPI-ImmTAAI in a more physiologically relevant cell system, we used the EndoC-β-cell line transduced with HLA-A*02. These pancreatic β cells naturally present the PPI_15–24_ peptide (**Supplementary Figures S5A, B**) and can activate NK and NK92 cells (51). We co-cultured NK92-PD-1 cells with EndoC-β cells for 4 h in the presence of ctr- or PPI-ImmTAAI (**Supplementary Figure S5A**; **Figures 5B, C**). The survival of the effector and target cells was not affected by ImmTAAI molecules binding to PD-1 or HLA-A*02-PPI (**Supplementary Figure S5C, D**). The targeted PD-1 agonist PPI-ImmTAAI inhibited NK92-PD-1 cell activation, as shown by a 54% decrease of the CD107a degranulation marker (**Figures 5B, C**, 10 nM ImmTAAI molecules). We subsequently co-cultured labelled EndoC-β cells with NK92-PD-1 to assess the impact on β-cell number over 70 h using Incucyte imaging. Co-culture with NK92-PD-1 led to a decrease in the number of live EndoC-β cells (**Supplementary Figures S5E–G**; **Figure 5D**, left). PPI-ImmTAAI protected EndoC-β cells (**Figures 5D–G**), whereas untargeted ctr-ImmTAAI provided no protection of EndoC-β cells in co-culture with NK92-PD-1 (**Figures 5D, F**). The IFN-γ concentration in the culture supernatant was also analysed after 70 h of co-culture. IFN-γ production was reduced in the presence of 0.1 nM PPI-ImmTAAI, with a maximal reduction of 48% achieved at 10 nM (**Figures 5G, H**).

A decrease in pancreatic β-cell number has a direct impact on pancreatic islet insulin production (68). To assess the effect of the PPI-ImmTAAI on insulin production, EndoC-β cells co-cultured for 70 h with NK92-PD-1 in the presence of increasing concentrations of ImmTAAI (**Figure 5D**) were challenged with glucose, and insulin was measured in the culture supernatants (**Figures 5I, J**). In the presence of PPI-ImmTAAI, EndoC-β cells produced significantly more insulin compared to cells incubated with ctr-ImmTAAI (**Figures 5I, J**). Taken together, we demonstrated that PPI-ImmTAAI can decrease NK92-PD-1 cytokine secretion and protect EndoC-β cells, whilst maintaining their capacity for insulin production.

### PPI-ImmTAAI modulates primary human PD-1^+^ NK-cell activation against EndoC-β cells

**Figure 6:**
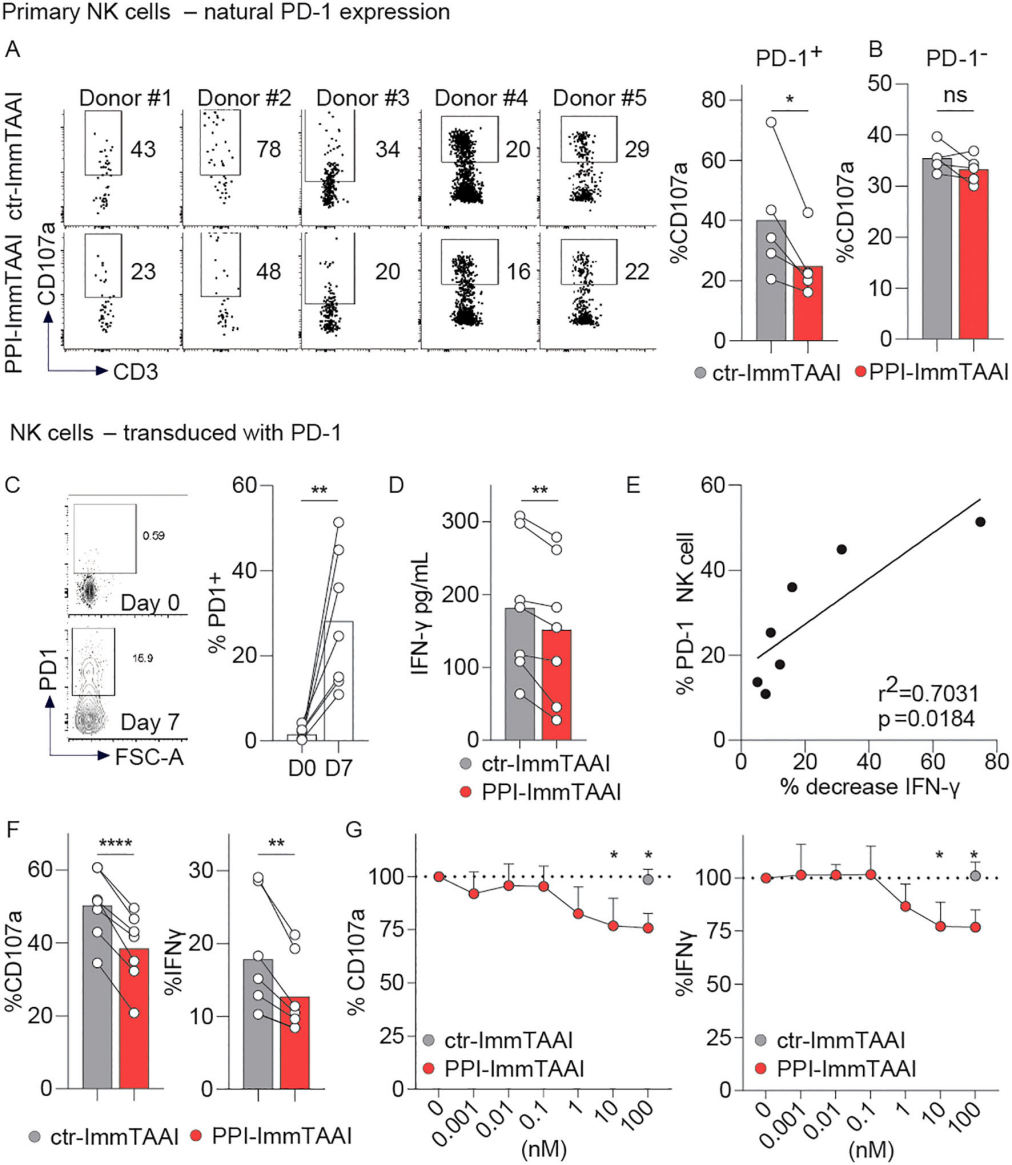
PPI-ImmTAAI inhibits human PD-1^+^ NK cells stimulated with EndoC-β cells. **(A, B)** Primary NK cells preactivated with IL-2 were stimulated for 4 h with EndoC-β cells in the presence of 10 nM ctr-ImmTAAI (grey) or 10 nM PPI-ImmTAAI (red). Representative flow cytometry profiles and graphs summarising CD107a expression in NK-PD-1^+^ **(A)** and NK-PD-1^−^ **(B)**. Each dot represents one sample (*n* = 5; mean; three independent experiments; paired *t*-test; ^*^ *p* ≤ 0.05). **(C–G)** PD-1-transduced NK cells were stimulated for 4 h with EndoC-β cells. **(C)** Preactivated NK cells were transduced with PD-1. Representative flow cytometry profiles and a graph summarising PD-1 expression at the indicated time point. Each dot represents one sample (mean; *n* = 7; three independent experiments; paired *t*-test; ^**^ *p* ≤ 0.01). **(D)** Graph summarising the IFN-γ concentration in the culture supernatant in the presence of 10 nM ctr-ImmTAAI (grey) or 10 nM PPI-ImmTAAI (red). Each dot represents one sample (mean; *n* = 7; three independent experiments; paired *t*-test; ^**^ *p* ≤ 0.01). **(E)** Graph showing the correlation between NK-cell PD-1 expression and IFN-γ decrease. Each dot represents one sample (*n* = 7; three independent experiments; simple linear regression; *r* ^2^ = 0.7031; *p* = 0.0184). **(F)** Graphs summarising CD107a expression and IFN-γ intracellular production by PD-1^+^ NK cells in the presence of 10 nM ctr-ImmTAAI (grey) or 10 nM PPI-ImmTAAI (red). Each dot represents one sample (*n* = 7; mean; three independent experiments; paired *t*-test; ^**^ *p* ≤ 0.01; ^****^ *p* ≤ 0.0001). **(G)** Graph summarising CD107a expression and IFN-γ intracellular production by PD-1^+^ NK cells in the presence of different concentrations of ctr-ImmTAAI (grey) or PPI-ImmTAAI (red), normalised to 0 nM ImmTAAI values. Each dot represents the mean of three independent experiments (*n* = 3; two-way ANOVA; SD; ^*^ *p* ≤ 0.05).

A small population of human circulating NK cells expresses PD-1 (**Supplementary Figure S6A**) (20–23). NK cells from five healthy donors were preactivated with IL-2 and co-cultured with EndoC-β cells for 4 h in the presence of ctr- or PPI-ImmTAAI. PD-1^+^ and PD-1^−^ primary NK cells were then analysed for CD107a degranulation marker expression (**Figures 6A, B**). PPI-ImmTAAI decreased PD-1^+^ NK-cell activation (**Figure 6A**) but had no effect on the PD-1^−^ population (**Figure 6B**).

To increase the percentage of NK cells expressing PD-1, we transduced primary NK cells with PD-1, allowing the PD-1^+^ population to increase from 1.5% to 28.1% (mean of seven donors, **Figure 6C**). Transduced NK cells were then activated for 4 h with EndoC-β cells (**Figures 6C–G**). Although there were a far greater number of PD-1^−^ than PD-1^+^ NK cells in the system, we observed a significant decrease in IFN-γ supernatant levels in the presence of PPI-ImmTAAI (**Figure 6D**) and found the proportion of IFN-γ decrease to be significantly correlated with the percentage of PD-1^+^ NK cells (**Figure 6E**). The effect of β-cell-bound PD-1 agonist molecules on PD-1^+^ and PD-1^−^ NK-cell populations was analysed separately by flow cytometry (**Figures 6F, G**; **Supplementary Figures S6B, C**). PPI-ImmTAAI had no effect on the PD-1^−^ NK-cell population (**Supplementary Figure S6B, C**), whereas PD-1^+^ NK-cell activation was significantly inhibited, as shown by the CD107a expression decrease from 50% to 38% (mean, **Figure 6F**), reaching a relative 25% reduction with 100 nM PPI-ImmTAAI (**Figure 6G**). IFN-γ intracellular production also decreased from 17% to 12% (mean, **Figure 6F**), reaching a relative 24% decrease with 100 nM PPI-ImmTAAI (**Figure 6G**). Thus, PPI-ImmTAAI decreased primary human PD-1^+^ NK-cell activation against a pancreatic β-cell line.

## Discussion

NK cells represent a key part of the immune response to viral pathogens, and their antitumour role is an area of active research (69). Additionally, the role played by NK cells in immune-mediated inflammatory diseases is increasingly appreciated (6, 8, 16), and modulating NK-cell activation could provide therapeutic benefit. Here, we showed for the first time that a targeted PD-1 agonist, ImmTAAI molecule can modulate PD-1^+^ NK-cell effector function and gene expression, allowing tissue-specific NK-cell suppression.

Autoimmune diseases result from the loss of self-tolerance by adaptive B and/or T cells responding against self. However, the network of immune cells involved in autoimmune disease biology also includes innate lymphoid cells, particularly NK cells, which act as a critical bridge between innate and adaptive immunity. Depending on the disease and its progression, NK cells may have a protective or pathogenic role (6–9, 12–17).

In murine models of T1D, the NK-cell contribution to T1D has been described by several groups. NK cells infiltrate the pancreas at an early stage of murine T1D development (37, 46), and disease progression is significantly delayed by NK-cell inhibition or depletion (46–48).

In humans, pancreatic NK-cell analyses in early-onset T1D patients are restricted by the availability of samples and the sensitivity of NK-cell detection techniques in tissues. However, NK-cell infiltration into the pancreas has been reported in T1D patients (70), and a second study has shown that the number of NK cells invading the pancreas in T1D patients is particularly increased in the context of enteroviral infection (45). This is noteworthy given the data linking viral infections to the triggering of T1D (71) and the role of NK cells in antiviral immunity. Several studies on circulating NK cells from T1D patients show that their phenotype evolves with disease progression (72–74). In young patients (75, 76) with recent-onset T1D or in adults with latent autoimmune diabetes, the circulating blood NK-cell population is reduced and activated compared to controls (72, 77). In long-standing T1D, NK cells have lower expression of both activating and inhibitory receptors, along with their corresponding ligands, compared to healthy controls (72, 78). Several *in vitro* studies have shown that human NK cells are activated when co-cultured with human pancreatic β cells (51, 79), and their cytokine production or cytotoxic effector functions could contribute to the inflammation and tissue destruction in T1D (49, 50, 80, 81). We reported that PPI-ImmTAAI decreases the killing of EndoC-β cells by the NK92-PD-1 cells. The protection of the EndoC-β cells is mainly due to the decrease in NK92-PD-1 cytotoxicity, but the effect of the PPI-ImmTAAI on NK92-PD-1 cytokine production also has a small but significant impact on EndoC-β-cell survival.

The PD-1 receptor is a major immune checkpoint expressed by various immune cell populations upon stimulation (82). The PD-1/PD-L1 pathway can modulate NK-cell activation (19, 83) and can be targeted by immunotherapies that block the PD-1/PD-L1 interaction to enhance NK-cell activation in the context of cancer (18, 28, 29, 31, 38–41). NK cells expressing PD-1 are found in the peripheral blood of healthy donors (20–23), and PD-1^−^ NK cells contain a pool of cytoplasmic PD-1 protein compatible with a rapid surface expression upon stimulation, highlighting the importance of this pathway in NK-cell function and regulation (21, 24). *In vitro*, human NK-cell PD-1 expression can be increased by cytokine and glucocorticoid stimulation (84, 85), and an increase in NK-cell PD-1 expression has been reported in human and mouse models of cancer, infection, and autoimmune diseases, including T1D (20, 25–37, 85–88). Thus, the PD-1 agonist effector portion of the ImmTAAI molecule has the potential to modulate the NK-cell compartment in these pathologies. We detected a decrease in primary NK-cell effector functions with the targeted PD-1 agonist molecule, even when only a small subset of the NK-cell population expressed PD-1, and we showed that pancreatic β-cell protection can preserve insulin production.

T1D is a disease area of high unmet clinical need, as current immunosuppressive treatments often show limited efficacy. The recent approval of teplizumab (Provention Bio, Sanofi, Paris, France), a T-cell-targeted therapy, is a promising step in T1D treatment (89). However, this systemic exposure to an anti-CD3 T-cell modulator could lead to serious side effects in young T1D patients and would not directly impact NK function. Our approach, which uses a tissue-targeted PD-1 agonist ImmTAAI molecule, has the potential to modulate not only T cells but also NK cells specifically in the localised setting, whilst avoiding systemic immunosuppression and the risk of immune-related side effects. We demonstrated that, in addition to PD-1^+^ effector T cells (42), tissue-bound PPI-ImmTAAI inhibits NK-cell activation, further strengthening its therapeutic potential. The effect of PPI-ImmTAAI on NK-cell modulation could represent an advantage compared to T-cell-specific therapies such as Teplizumab, or systemic anti-PD-1^−^ agonist therapies (rosnilimab; AnaptysBio, and peresolimab; Lilly, Indianapolis, Indiana, US), as we demonstrated that the soluble PD-1 agonist had no effect on NK-cell inhibition in our systems.

In this study, we show for the first time that a targeted PD-1 agonist modifies NK-cell effector function and transcriptional profile in the context of T1D. The ImmTAAI molecule, in its unbound form, is inactive, thereby avoiding systemic T- and NK-cell immunosuppression and reducing the risk of long-term immune-related side effects or impact on tumour surveillance. The role of NK cells in numerous autoimmune pathologies is underappreciated, and we propose that localised ImmTAAI-mediated NK-cell inhibition could be an important aspect alongside T-cell modulation in the context of T1D disease modification.

## Data Availability

The datasets presented in this study can be found in online repositories. The names of the repository/repositories and accession number(s) can be found below: https://www.ncbi.nlm.nih.gov/, GSE288132.

## Supplementary material

Supplementary Figure 1 related to **Figure 1**: ImmTAAI molecule. Graphic representation of the ImmTAAI molecule. ImmTAAI is comprised of an affinity enhanced targeting domain, which binds to the target epitope with picomolar affinity ➀, fused to a PD-1 agonist moiety ➁, with an Fc fragment for half-life extension ➂.

Supplementary Figure 2 related to **Figure 1**: Triggering PD-1 pathway inhibits NK92-PD-1 activation. **(A)** Representative flow cytometry profiles and graphs summarizing the level of expression of PD-1 by NK92 cell line and PD-L1 by K562 cell line (each dot represents one sample, *n* = 3–5, mean, 3 independent experiments, t-test, ****p ≤ 0.0001). **(B)** Representative flow cytometry profile and graph summarizing the number of irrelevant-ImmTAAI molecules (specific for HLA-A*02-irrelevant peptide, grey dot) and PPI-ImmTAAI molecules (black dots) used at the indicated concentration to bind to K562-HLA-A*02 loaded with PPI peptide (each dot represents the mean, SD, representative of 3 independent experiments). **(C)** NK92-PD-1 cells stimulated for 4 h with K562-HLA-A*02-PPI in the presence of ctr-ImmTAAI (grey) or PPI-ImmTAAI (red). Representative flow cytometry profiles and graphs summarizing proportion of dead NK92-PD-1 in the presence of different ImmTAAI molecules concentration. (Each dot represents the mean of 2 independent experiments, *n* = 4, two-way ANOVA, SD). **(D)** NK92-PD-1 cells were stimulated for 20 h with K562-HLA-A*02-PPI in the presence of ctr-ImmTAAI (grey) or PPI-ImmTAAI (red). Graph summarizing proportion of dead NK92-PD-1 cells in the presence of different ImmTAAI concentrations. (Each dot represents the mean of 2 independent experiments, *n* = 4, two-way ANOVA, SD). **(E)** K562-HLA-A*02-PPI were cultured for 24 h in presence of ctr-ImmTAAI (grey) or PPI-ImmTAAI (red). Graph summarizing the proportion of live K562-HLA-A*02-PPI cells in the presence of different ImmTAAI concentrations. (Each dot represents the mean of 2 independent experiments, *n* = 4, two-way ANOVA, SD). (F-G) NK92-PD-1 cells stimulated for 4 h with K562-HLA-A*02-PPI without ImmTAAI (black) or with 10nM ctr-ImmTAAI (grey). **(F)** Graphs summarizing CD107a expression and IFN-γ intracellular production (each dot represents one sample, *n* = 6, mean, 3 independent experiments, paired *t*-test). **(G)** Graphs summarizing IFN-γ, TNF-α, and GM-CSF concentration in the culture supernatants of activated NK92-PD-1 (each dot represents one sample, *n* = 6, mean, 3 independent experiments, paired *t*-test).

Supplementary Figure 3 related to **Figure 3**: PPI-ImmTAAI binding to K562-HLA-A*02 not pulsed with PPI peptide. **(A)** Representative flow cytometry profile summarizing the binding of PPI-ImmTAAI-AF488 to K562-HLA-A*02, loaded (red) or not (blue) with PPI peptide. (2 independent experiments). **(B)** Microscopy images of co-cultured K562-HLA-A*02 not pulsed with PPI peptide (red and blue double positive) with NK92-PD-1 (blue) in the presence of PPI-ImmTAAI-AF488 (green). (2 independent experiments).

Supplementary Figure 4 related to **Figure 4**: Isolation of NK92-PD-1 cells activated or not with K562-HLA-A*02-PPI in the presence of ctr- or PPI-ImmTAAI. (A-F) Unstimulated NK92-PD-1 cells (black) or stimulated for 20 h with K562-HLA-A*02-PPI in the presence of ctr-ImmTAAI (grey) or PPI-ImmTAAI (red). **(A)** Representative flow cytometry profiles of activated NK92-PD-1 before and after sort (4 independent experiments, *n* = 4). **(B)** Graph summarizing the proportion of isolated live NK92-PD-1 (each dot represents one sample, *n* = 4, mean, 4 independent experiments, one-way ANOVA). **(C-E)** Graphs summarizing IFN-γ, TNF-α, and GM-CSF concentrations in the culture supernatants for each experiment (each bar represents one sample, *n* = 4, mean, 4 independent experiments). **(F)** Geneset enrichment analysis (GSEA) results showing the normalised enrichment scores (NES) for all genesets from the Immunologic collection that refer to NK cells in the geneset title. Number of genes in geneset represented by dot size, and –log10(padj) represented by colour scale.

Supplementary Figure 5 related to **Figure 5**: EndoC-β cell killing. **(A)** Graphic representation of NK92-PD-1 activation with EndoC-β cells in the presence of PPI-ImmTAAI. **(B)** Representative flow cytometry profile and graph summarizing the number of irrelevant-ImmTAAI (specific for HLA-A*02-irrelevant peptide, with dot) and PPI-ImmTAAI (black dot) used at the indicated concentration to bind to EndoC-β cells (each dot represents the mean, SD, representative of 3 independent experiments). **(C)** NK92-PD-1 cells stimulated for 4 h with EndoC-β cell in presence of ctr-ImmTAAI (grey) or PPI-ImmTAAI (red). Representative flow cytometry profiles and graphs summarizing proportion of dead NK92-PD-1 cells in the presence of different ImmTAAI molecules concentrations. (Each dot represents the mean of 2 independent experiments, *n* = 4 two-way ANOVA, SD). **(D)** EndoC-β cells were cultured for 72 h in the presence of ctr-ImmTAAI (grey) or PPI-ImmTAAI (red) or without ImmTAAI (black). The left graph represents the ratio of EndoC-β cells number overtime, normalized to 0h and in the presence of 100nM ImmTAAI molecules or no ImmTAAI (representative of 2 independent experiments, SD). The right graph summarizes the proportion of live EndoC-β cells in the presence of different concentration of ImmTAAI molecules and normalized to 0nM ImmTAAI values. (Each dot represents the mean of 2 independent experiments, *n* = 4, mean, two-way ANOVA, SD). **(E-G)** EndoC-β cells expressing a red nuclear fluorophore were cultured for 70h in the presence or absence of NK92-PD-1. (E) Representative microscopy images of EndoC-β cell (red) alone (left panels) or co-cultured with NK92-PD-1 (right panels) at 0 (top panels) or 70 h (bottom panels) (representative of 5 independent experiments). **(G)** Graph representing the ratio of EndoC-β cells number overtime, normalized to 0h and cultured in the presence (blue) or absence (black) of NK92-PD-1 cells (representative of 5 independent experiments, SD). **(H)** Graphs summarizing the proportion of live EndoC-β cell decrease when cultured alone (black, 0% decrease) or with NK92-PD-1 (blue) (each dot represents one sample, *n* = 8, mean, 4 independent experiments, paired *t*-test, ****p ≤ 0.0001).

Supplementary Figure 6 related to **Figure 6**: PPI-ImmTAAI inhibit human PD-1^+^ NK cells stimulated with EndoC-β cells. **(A)** Representative flow cytometry profiles and graph summarizing PD-1 expression by resting NK cells (each dot represents one donor, *n* = 25, 10 independent experiments). **(B, C)** PD-1 transduced NK cells were stimulated for 4 h with EndoC-β cells. **(B)** Graphs summarizing CD107a expression and IFN-γ intracellular production by PD-1^-^ NK cells in the presence of 10nM ctr-ImmTAAI (grey) or 10 nM PPI-ImmTAAI (red) (each dot represents one sample, *n* = 7, mean, 3 independent experiments, paired *t*-test). **(C)** Graph summarizing CD107a expression and IFN-γ intracellular production by PD-1^-^ NK cells in the presence of different concentration of ctr-ImmTAAI (grey) or PPI-ImmTAAI (red) and normalized to 0nM ImmTAAI values. (Each dot represents the mean of 3 independent experiments, *n* = 7, two-way ANOVA, SD).
