# Supplementary material for: Cell-targeted PD-1 agonists are potent NK-cell inhibitors
Source: Front Immunol. 2025 Aug 22;16:1640509. doi: 10.3389/fimmu.2025.1640509 (PMC12412223; doi:10.3389/fimmu.2025.1640509)
Supplement: Supplementary Figure 1 — related to Figure 1 : ImmTAAI molecule. Graphic representation of the ImmTAAI molecule. ImmTAAI is comprised of an affinity enhanced targeting domain, which binds to the target epitope with picomolar affinity ➀, fused to a PD-1 agonist moiety ➁, with an Fc fragment for half-life extension ➂. [file DataSheet1.pdf]

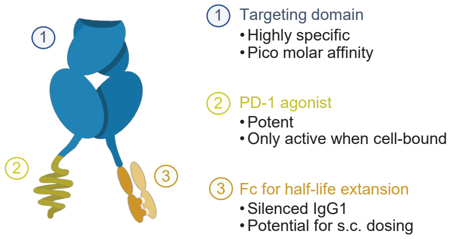

Supplementary Figure 1 related to Figure 1: ImmTAAI molecule

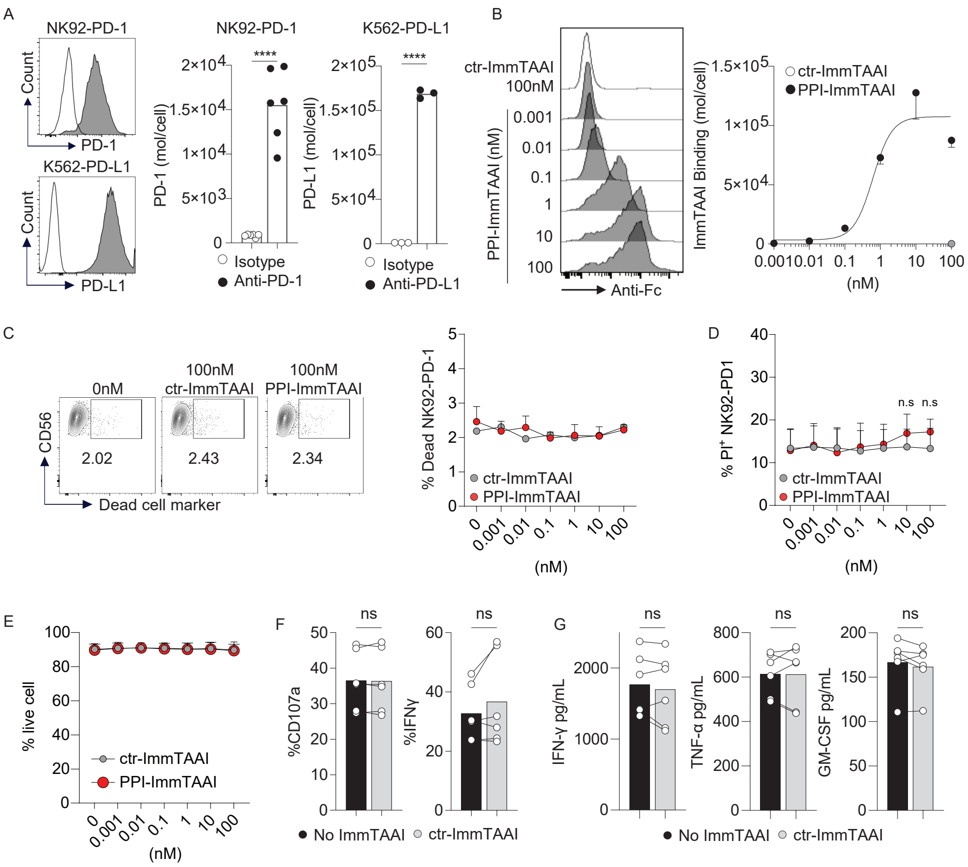

Supplementary Figure 2 related to Figure 1: Triggering PD-1 pathway inhibit NK92-PD-1 activation.

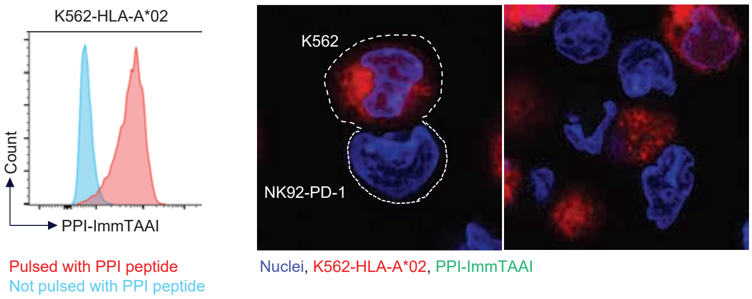

Supplementary Figure 3 related to Figure 3: PPI-ImmTAAI binding to K562-HLA-A*02.

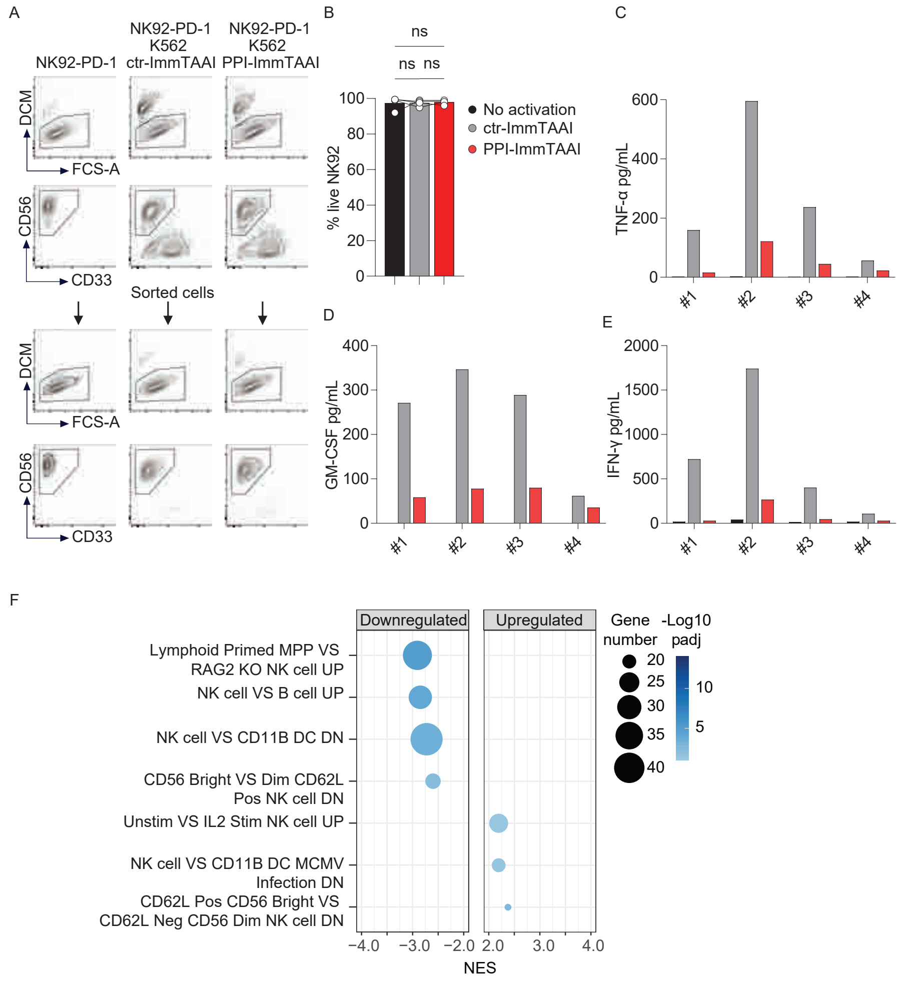

Supplementary Figure 4 related to Figure 4: Isolation of NK92-PD-1 cells activated or not with K562-HLA-A*02-PPI in presence of ctr- or PPI-ImmTAAI.

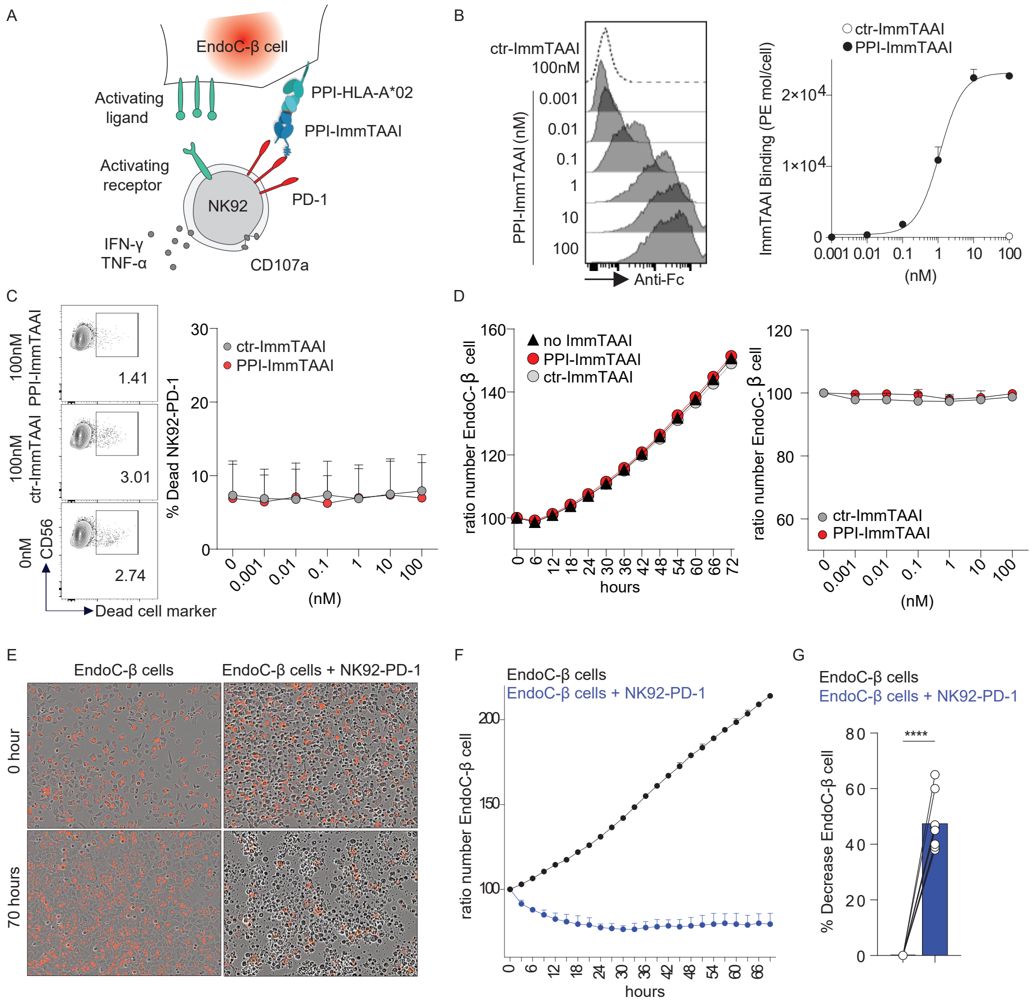

Supplementary Figure 5 related to Figure 5: EndoC-β cell killing.

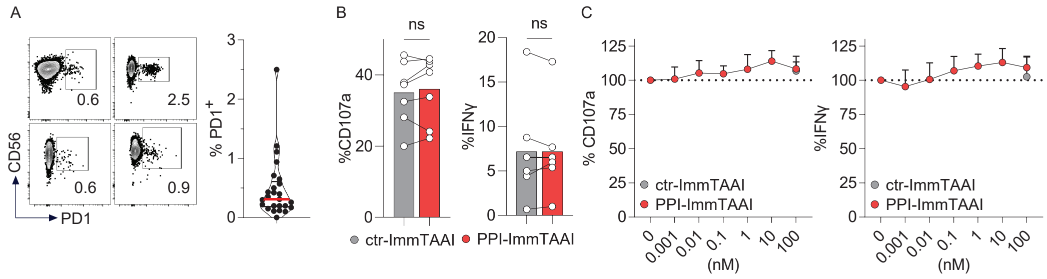

Supplementary Figure 6 related to Figure 6: PPI-ImmTAAI inhibit huamn PD-1- NK cells stimulated with EndoC-β cells.
